# *In silico* and *in vivo* evaluations of multistage antiplasmodial potency and toxicity profiling of n-Hexadecanoic acid derived from *Vernonia amygdalina*

**DOI:** 10.3389/fphar.2024.1445905

**Published:** 2024-08-21

**Authors:** F. I. D. Afolayan, R. A. Odeyemi, R. A. Salaam

**Affiliations:** Department of Zoology, University of Ibadan, Ibadan, Nigeria

**Keywords:** molecular docking, n-hexadecanoic acid, *Plasmodium berghei*, ADMET, antiplasmodial activity, chemosuppression, toxicity test

## Abstract

**Background:**

Despite the widely reported potentials of n-Hexadecanoic acid (HA) as a bioactive, its multi-stage antiplasmodial activity and toxicity profiles remain largely unknown.

**Methodology:**

Thus, this study uses a combination of *in silico* approaches and *in vivo* studies to assess the inhibitory activities of HA at different stages of the Plasmodium lifecycle, antiplasmodial performance, and toxicity profiles. The HA was retrieved from the PubChem database, while antiplasmodial target proteins from different stages of the *Plasmodium falciparum* life cycle were collated from the Protein Databank (PDB). Molecular Docking and Visualization were conducted between the compound and target proteins using AutoVina PyRx software and Biovia Discovery Studio, respectively. Also, the AdmetLab 3.0 algorithm was used to predict the absorption, Distribution, Metabolism, Excretion, and Toxicity (ADMET) profiles of HA. Based on a 4-day suppressive test, the antiplasmodial activity against the *Plasmodium berghei ANKA* strain in mice was evaluated. Furthermore, subacute toxicity and micronucleus assays were used for further toxicity assessment.

**Results:**

The molecular docking analysis indicates multi-stage, multi-target potentials of HA with favourable ligand-receptor complexes across the four *Plasmodium falciparum* stages. Meanwhile, the mice administered with 100 mg/kg, 50 mg/kg, and 10 mg/kg of HA demonstrated considerable chemosuppression in a dose-dependent manner of 89.74%, 83.80%, and 71.58% percentage chemosuppression, respectively, at *p* < 0.05. The ADMET prediction, histopathological tests, and micronucleus assays show that HA is safer at a lower dose.

**Conclusion:**

This study showed that n-Hexadecanoic acid is a potential drug candidate for malaria. Hence, it is recommended for further molecular and biochemical investigations.

## 1 Introduction

Malaria is a protozoan infection that causes an intense fever and is spread to people by female Anopheles mosquitoes that have been infected with it (White et al., 2014). Human malaria can be caused by any of the five species of the Plasmodium genus, especially *Plasmodium falciparum;* being the deadliest strain and most common in Africa (World Health Organization, 2018). While *Plasmodium vivax* is less virulent than *Plasmodium falciparum*, it can persist in the body for weeks or months in a dormant form. This can lead to relapses. However, *Plasmodium vivax* remains the predominant malaria parasite in the majority of nations besides sub-Saharan Africa. Thus, a fight against the *Plasmodium spp*. is paramount in a bid to eradicate malaria disease.

According to the World Health Organization, almost half of the world’s population is at risk of malaria (World Health Organization, 2022). Among the groups of people at high risk of contracting malaria and developing the severe disease are infants, children under 5 years of age, pregnant women, and patients with HIV/AIDS (World Health Organization, 2024). People who migrate to areas with intense malaria transmission and low immunity are also susceptible to the attack of this disease, in which over 90% of deaths occurred in Africa. As of 2022, about 249 million people in 85 nations were at risk of Malaria, with the health challenge claiming the lives of about 608,000 individuals (World Health Organization, 2019).

The burden of malaria continues to fall disproportionately heavily on the African continent. The majority of malaria cases were found in the following African nations in 2021 due to a rise in malaria prevalence: Nigeria (39.0%), the Democratic Republic of the Congo (18.2%), Uganda (7.8%), and Mozambique (6.1%) (World Health Organization, 2024). The continuous devastating effects of malaria on the world at large and in Nigeria are indeed worrisome. This is because malaria, a condition that poses a serious risk of death and accounts for millions of clinical cases, has an adverse effect on both individuals and society (World malaria report, 2010). Sadly, most currently available antimalarial drugs are becoming less effective due to the emergence and spread of parasites that are resistant to the drugs (Ouji et al., 2018), which hinders clinical trials and patient compliance. Thus, it becomes necessary to seek novel antiplasmodial agents against Plasmodium parasites.

Although the malaria vaccine is currently available in some countries, it is not available in most of the endemic regions (Snounou et al., 2005). Thus, anti-malarial medications that can kill parasites inside the body are still very much the most effective means of treatment (Snow et al., 2005). As the principal approach for treating *P. falciparum* malaria and reducing the danger of resistance, artemisinin-based combination treatment (ACT) has been endorsed by the World Health Organization (WHO). This burden is worsening despite the availability of standard antimalarial medications, primarily because *Plasmodium falciparum* is evolving a greater resistance to the commonly prescribed antimalarial medications. The use of sesquiterpene lactone (Artemisinin) and its metabolites are being used as antimalarial drugs, which prompts the investigative research of antimalarial activity in other terpenoids. Plant extracts and phytochemicals have been extensively utilized in the production of various drugs, including the presently available antimalarial drugs, and therefore could be thought of as a possible source of novel antimalarial drugs with possible immunostimulatory potentials (Misganaw et al., 2020).

*Vernonia amygdalina*, also known as the bitter leaf, a tiny perennial shrub with dark green leaves and coarse barks found predominantly in tropical Africa, has been reported to contain different phytochemicals that possess antimalarial properties (Afolayan and Abdulkareem, 2021). It has been traditionally used as a potent malaria regimen as well as a treatment for various parasitic infections, gastrointestinal problems, amoebic dysentery, bacterial infection, and inflammatory diseases (Afolayan and Abdulkareem, 2021; Afolayan and Sowemimo, 2022). These potencies and ethnobotanical usage have been linked to some of its bioactive components. The research carried out on *Vernonia amygdalina* by Afolayan and Abdulkareem (Afolayan and Abdulkareem, 2021) in an *in silico* study reported that Phytol and n-Hexadecanoic acid (HA) are the most abundant phytochemical constituents that show high antimalarial and anti-inflammatory potentials.

However, the specific stage of *Plasmodium spp.* lifecycle in which the bioactive can have significant positive impacts remains unknown. Also, the antimalarial activity of HA on malaria parasites *in vivo* still needs proper investigation, including its toxicity state.

The n-Hexadecanoic acid, also known as Palmitic acid (PA), is a fatty acid that contains a 16-carbon chain. It is chemically written as CH_3_ (CH_2_)_14_COOH. Palmitic acid is the most common saturated fatty acid in animals, plants, and microorganisms (Carta et al., 2017). The most prevalent saturated fatty acid in the human body is palmitic acid, which can either be consumed by food or produced naturally in the body from other fatty acids, carbohydrates, and amino acids. In membrane phospholipids (PL) and adipose triacylglycerol (TAG), palmitic acid makes up 20%–30% of the total fatty acids (FA) (Carta et al., 2015). As the name suggests, palmitic acid makes up a considerable portion of palm oil (44% of total fats), but also present in meat and dairy products (50%–60% of total fats), cocoa butter (26%) and olive oil (8%–20%) in significant levels. Furthermore, palmitic acid is present in breast milk with 20%–30% of total fats (Innis, 2016). The average intake of PA is around 20–30 g/day (Sette et al., 2011). n-Hexadecanoic acid has been reported to exhibit potential antioxidant and anticancer properties (Bharath et al., 2021). It inhibits the proliferation of cancer cells by inducing apoptosis and cell cycle arrest. Also, it has been reported to have cytotoxic effects on cancer cells, induces the proliferation of bone marrow mesenchymal stem cells (Chen et al., 2010; Ravi and Krishnan, 2016; Bharath et al., 2021), and act as an antifungal (Abubacker and Deepalakshmi, 2013) and antibacterial.

Given the remarkable potentials of n-Hexadecanoic acid as a bioactive, this study investigated the lifecycle stage of *Plasmodium spp*., in which n-Hexadecanoic acid has the most significant antiplasmodial activity, determine its chemosuppression performance and assess its toxicity potential *in silico* and *in vivo*. Thus, a combined approach of molecular docking, computational ADMET prediction, 4-day suppressive test, and subacute toxicity test were employed to achieve the research objectives.

## 2 Materials and methods

### 2.1 *In silico* investigations

#### 2.1.1. Retrieval of the n-Hexadecanoic acid compound

Following our previous work, which identified n-Hexadecanoic acid (HA) as an antiplasmodial bioactive from multiple herbal plants, including *Vernonia amygdalina* (Afolayan and Abdulkareem, 2021), the structured data file (SDF) of the compound was downloaded from the PubChem database (https://pubchem.ncbi.nlm.nih.gov/) with CID: 985.

#### 2.1.2 Collection of *Plasmodium falciparum* protein targets

For this study, eight proteins identified in four different stages of the Plasmodium lifecycle were retrieved from the PDB (https://www.rcsb.org/) database containing experimentally determined structures of the proteins in 3D and computed structure models from AlphaFold DB and ModelArchive. These proteins belong to four stages of the Plasmodium lifecycle, including (i) Asexual Blood Stage: Calcium-independent protein kinases (CDPK) (PDB ID: 4QOX), Purine nucleoside phosphorylase (PNP) (PDB ID: 2BSX), and Dihydrofolate reductase-thymidylate synthase (DHFR-TS) (PDB ID: 3DGA); (ii) Hepatic Schizonts stage (Liver Prophylaxis): Enoyl-ACP Reductase (ENR) (PDB ID: 2FOI), and M1 alanyl aminopeptidase (A-M1) (PDB ID: 4X2U); (iii) Transmission stage 1 (targeting parasite gametocytes): Gametocyte surface protein 45/48 (s48/45) (PDB ID: 7ZWF) and Cysteine-rich 230 kDa gamete surface protein (PF230) (PDB ID: 7USS); (iv) Transmission stage 2 (targeting the insect vector): Protein kinase 7 (PK7) (PDB ID: 2PML) (Salaam and Afolayan, 2024).

#### 2.1.3 Identification of active sites of *Plasmodium falciparum* protein targets

The active sites of the experimentally determined protein were identified using the Computed Atlas of Surface Topography of protein 3.0 (Castp 3.0) (http://sts.bioe.uic.edu/castp/). Out of the eight proteins, six proteins with PDB ID: 2PML, 3DGA, 4RGJ, 2BSX, 4X2U, and 2FOI were identified and reconfirmed using literature, while two proteins with PDB ID: 7USS and 7ZWF were unavailable. Thus, the first six proteins were subjected to site-directed docking, while the latter two proteins were subjected to a blind docking process.

#### 2.1.4 Preparation of *Plasmodium falciparum* target proteins

All eight proteins were uploaded on Biovia Discovery Studio Visualizer software (https://discover.3ds.com/discovery-studio-visualizer-download) for the first stage of preparation. At this stage, the heteroatoms and any existing ligands were removed by exploring the components of each protein’s hierarchy. The second stage of the preparation was completed using the UCSF Chimera (https://www.cgl.ucsf.edu/chimera/download.html). The DockPrep tool on Chimera enabled the replacement of missing charges, the addition of hydrogen, and the inclusion of Gaisteger charges. These processes helped prevent any interference during the docking process (Tarkaa et al., 2023).

##### 2.1.5 Assessment of binding affinity with molecular docking simulation

A molecular docking simulation was performed to determine the binding affinity of the HA and each of the proteins. This also enables the visualization of the complexes to study the interaction and possible stability and flexibility of the interaction. To perform the simulation, PyRx, a virtual screening tool, was employed by using the AutoDock Vina integrated into the system. The ligand (HA) was loaded using the “Chemical Table File” via “Import.” Meanwhile, each protein was loaded as a molecule and made into a macromolecule for the docking process. After the minimization and conversion of the ligand into PDBQT format, the docking process was initiated (Abdelmonsef et al., 2022).

##### 2.1.6 Visualization of coupled ligand-receptor complex

After a successful docking process, the HA was coupled with each of the proteins to visualize the interaction. In this aspect, Biovia Discovery Studio Visualizer software was employed to study the complexes in 3D and 2D visuals (Afolayan F. I. D. et al., 2023a).

##### 2.1.7 Absorption, distribution, metabolism, excretion, and toxicity (ADMET) analysis

The analysis of selected *in silico* tests for the Absorption, Distribution, Metabolism, Excretion, and Toxicity properties of HA was conducted using the latest ADMETLAB 3.0 database (https://admetlab3.scbdd.com/). This helps understand the drug’s (un) desirable pharmacokinetic, pharmacodynamics, and toxicity qualities to avoid late attrition as much as possible. Also, the physicochemical properties of the ligand were predicted using the database, which gave insight into the pharmaceutical friendliness and drug-likeness properties of the HA.

### 2.2 *In vivo* investigations

#### 2.2.1 Materials

n-Hexadecanoic acid was purchased from Chemscene LLC (Princeton, United States), while laboratory mice were purchased from Loozap farm, Ibadan, Nigeria. The reagents for vehicle were obtained from the Cell Biology and Genetics Laboratory (Zoology Department, University of Ibadan), while consumable materials were bought from Juliemak stores, Yemetu, Ibadan.

#### 2.2.2 Animals

A total number of sixty (60), 6–8 weeks old laboratory albino mice (*Mus musculus*) were obtained from Loozap Farms, Ibadan, Nigeria. They were weighed and examined for fitness before the commencement of the experiment. The animals were caged in groups (n = 5), at the Animal House facility, Department of Zoology. They were acclimatized to the housing condition for 2 weeks before the commencement of the experiment. Feeding and drinking of water were done *ad libitum* to ensure maximum consumption and optimum condition.

#### 2.2.3 Parasite

*Plasmodium berghei ANKA* was obtained from the Institute of Advanced Medical Research and Training (IMRAT), College of Medicine, University of Ibadan, and maintained by serial passage according to the protocol described by Nyandwaro et al., (2020).

#### 2.2.4 Assessment of antiplasmodial activities

##### 2.2.4.1 Inoculation of parasite

Twenty-five (25) albino mice (weighing 22 ± 2 g) were randomly divided into five (5) groups of five animals per group (n = 5). Parasitized blood was obtained from infected donor mouse with rising parasitaemia (≥ 20%) by cardiac puncture and the infected blood was diluted with phosphate saline glucose buffer (PSG-mouse) to attain an estimate of 5 × 10^7^ red blood cells per ml. Then, 0.2 mL of inoculum containing 1 × 10^7^ infected RBCs was injected into the abdominal cavity of experimental animals by intraperitoneal (ip) injection.

##### 2.2.4.2 Drug Administration

The groups 1, 2, and 3 were administered 10 mg/kg, 50 mg/kg, and 100 mg/kg of HA, respectively, while groups 4 and 5 were administered Artemether/lumefantrine and the vehicle, respectively. All drugs were administered orally for 4 days.

##### 2.2.4.3 Suppressive test

The chemo-suppressive effect of HA in mice infected with *P. berghei* was assessed using Peter’s 4-day suppressive test. The mice were infected with the malaria parasite inoculum (1 × 10^7^ *P.berghei* ANKA) on the first day of the experiment (D0) and treated as indicated in the section above on Animal Grouping and Drug Administration after 2 h post-infection. The treatment continues for another three consecutive days. Each mouse’s parasitaemia level was assessed 96 h after infection (D4). Animals in each group had their PCV, rectal temperature, and body weight measured both before and after the experiment.

##### 2.2.4.4 Determination of parasitaemia, percentage chemo-suppression, packed cell volume, rectal temperature, and body weight

Parasitaemia was determined by counting the number of infected RBCs (minimum of four fields per slide) using a light microscope with an objective lens magnification power of 1,000x. Percentage parasitaemia and percentage chemo-suppression were calculated using the modified Peters and Robinson formula (Nyandwaro et al., 2020):

$$\%\;parasitemia=\frac{number\;of\;parasitized\;RBCs}{total\;number\;of\;RBC\;count}\times 100$$

$$\%\;chemo-suppression=\frac{\%\;parasitemia\;in\;negative\;control-\;\%\;parasitemia\;in\;study\;group}{\%\;parasitemia\;in\;negative\;control}\times 100$$

Blood samples were taken from each mouse’s tail using heparinized capillary tubes, sealed, and then put into a micro haematocrit centrifuge to calculate the packed cell volume of the blood. After being centrifuged for 5 min at 12,000 rpm, the blood samples’ PCV was calculated using the following formula:

$$Packed\;Cell\;Volume=\frac{volume\;of\;erythrocyes\;in\;a\;given\;volume\;of\;blood}{total\;volume\;of\;blood}\times 100$$

The body weight and rectal temperature of each mouse were also determined using a sensitive digital weighing balance and rectal thermometer, respectively.

##### 2.2.4.5 Determination of mean survival time

The survival rate was monitored from the first day of the treatment to 30 days. Each mouse’s mortality was monitored and recorded in days. The mean survival time (MST) of mice in each group was determined, as shown below.

$$\text{MST}=\frac{sum\;of\;survival\;time\;of\;all\;mice\;in\;a\;group\;in\;days}{total\;number\;of\;mice\;in\;a\;group}$$

#### 2.2.5 Subacute toxicity test

Thirty mice were grouped into six (n = 5) and treated orally with HA for 14 days. The negative control group (group a) was treated with 0.2 mL of the vehicle, while the positive control group (group g) was also treated with the 20 mg/kg dosage of cyclophosphamide. Meanwhile, groups b, c, d, e, and f were treated with 2 mg/kg, 5 mg/kg, 10 mg/kg, 50 mg/kg, and 100 mg/kg of HA, respectively. The blood samples were collected for haematological analysis, while the liver and kidney were also collected for histopathological analysis and fixed in 10% formalin.

##### 2.2.5.1 Histopathological study

The tissues were sliced into small pieces not more than 4 mm thick and then submerged in 10% formal saline for 24 h to fix, according to the protocols of Avwioro et al., (2010) for histopathology investigations. The tissue processing was carried out automatically using an automatic tissue processor (Leica TP 1020) by letting the tissues pass through several reagents: 10% formal saline was used in Stations 1 and 2, and alcohol in Stations 3 through 7 was used to dehydrate the tissues. The tissues were then moved into Stations 10 to 12, which contained wax for infiltration or impregnation, after passing through Stations 8 and 9, which contained xylene for the purpose of cleaning. Tissues stayed in each station for 1 hour during the 12-h duration of these operations. The tissues were then covered with molten paraffin wax, allowed to cool on ice, and then sectioned at a 4-micrometer interval. These parts were picked up using clean, labeled slides and floated in a water bath set at 55°C. The slides were then dried for an hour on a hotplate at 60°C. Haematoxylin and Eosin staining were applied to the slides, which were then dried and examined under a light microscope.

##### 2.2.5.2 Micronucleus (MN) assay

The animals used for the subacute toxicity test were also used for the MN assay because the treatment follows the same procedure, but the positive control, cyclophosphamide (group g) for MN, is a single-dose treatment that was given intraperitoneally. The MN assay was carried out as described by Jain and Pandey (Jain and Pandey, 2019), four animals were sacrificed from each group by cervical dislocation, and both femurs were removed. The epiphyses were removed from the femur, and the bone marrow was flushed with fetal bovine serum (FBS) into Eppendorf tubes (1.5 mL). After a light tap to ensure appropriate cell dispersion, it was centrifuged at 2,000 rpm for 5 minutes. The pellet was suspended in another 1 mL of FBS in the Eppendorf tube, thoroughly mixed using a micropipette, and centrifuged once more at the same rate after the supernatant was discarded. Once more, the supernatant was removed, and the pellet was mixed with 0.5 mL of FBS before a few drops of the viscous mixture were dropped on a clean, oil-free slide. This drop was spread on the slide and allowed to air dry.

The slides were stained with 0.4% May-Grunewald stain for 3-4 min and were immediately transferred into another coupling jar containing May-Grunewald and distilled water (ratio 1:1) and allowed to stain for another 3-4 min. The slides were rinsed in distilled water and allowed to dry completely. Then, it was also stained in 5% Giemsa stain for 5 min, rinsed in distilled water, and allowed to dry completely. A minimum of 1,000 cells from each mouse were examined for micronuclei in polychromatic erythrocytes (MNPCE) using a light microscope at a magnification of 1,000X (oil immersion). The relative size of the erythrocytes and the difference in staining between PCEs (bluish-purple) and normochromatic erythrocytes (NCEs, pink-orange) are used to distinguish between them.

### 2.3 Statistical analysis

Data analysis was done using One-way ANOVA (Analysis of variance) followed by Dennett’s Multiple Comparison Test with the aid of Graph Pad Prism (Version 5.0, Graph Pad Software Inc., La Jolla, California, and United States). The results were expressed as the standard error of the mean (SEM), and a *P*-value of ≤ 0.05 was used to determine its level of significance.

## 3 Results

### 3.1 Molecular docking analysis outcome

The compound, n-Hexadecanoic acid (HA), interacted with the selected *Plasmodium spp*. protein targets. However, the binding affinities of the interaction vary, as shown in Table 1. The binding affinities indicate that the ligand is capable of interacting with multiple antiplasmodial targets, notably Enoyl-ACP Reductase, Protein kinase 7, M1 alanyl aminopeptidase, Calcium-independent protein kinases, and Gametocyte surface protein with binding affinities at least or lower than −5.0 Kcal/mol.

**TABLE 1:** Binding Affinity scores of n-Hexadecanoic acid against multiple Protein Targets of *Plasmodium spp*.

		Target	Binding affinity (Kcal/mol)					
Compound	PF-PNP	PF-ENR	PF-PK7	PF-A-M1	PF-CDPK4	PF-DHFR-TS	PF-S230	PF-S48/45
n-Hexadecanoic acid	−4.7	−5.8	−5.0	−5.5	−5.6	−4.0	−4.5	−5.6

PF-CDPK4: *Plasmodium falciparum* Calcium-independent protein kinases; PF-DHFR-TS: *Plasmodium falciparum* Dihydrofolate reductase-thymidylate synthase; PF-PNP: *Plasmodium falciparum* purine nucleoside phosphorylase; PF-S48/45: *Plasmodium falciparum* Gametocyte surface protein; PF-S230: *Plasmodium falciparum* Cysteine-rich 230 kDa gamete surface protein; PF-PK7: *Plasmodium falciparum* Protein kinase 7; PF-ENR: *Plasmodium falciparum* Enoyl-ACP, reductase; PF-A-M1: *Plasmodium falciparum* alanyl aminopeptidase M1.

### 3.2 Ligand-receptor complex analysis

**FIGURE 1:**
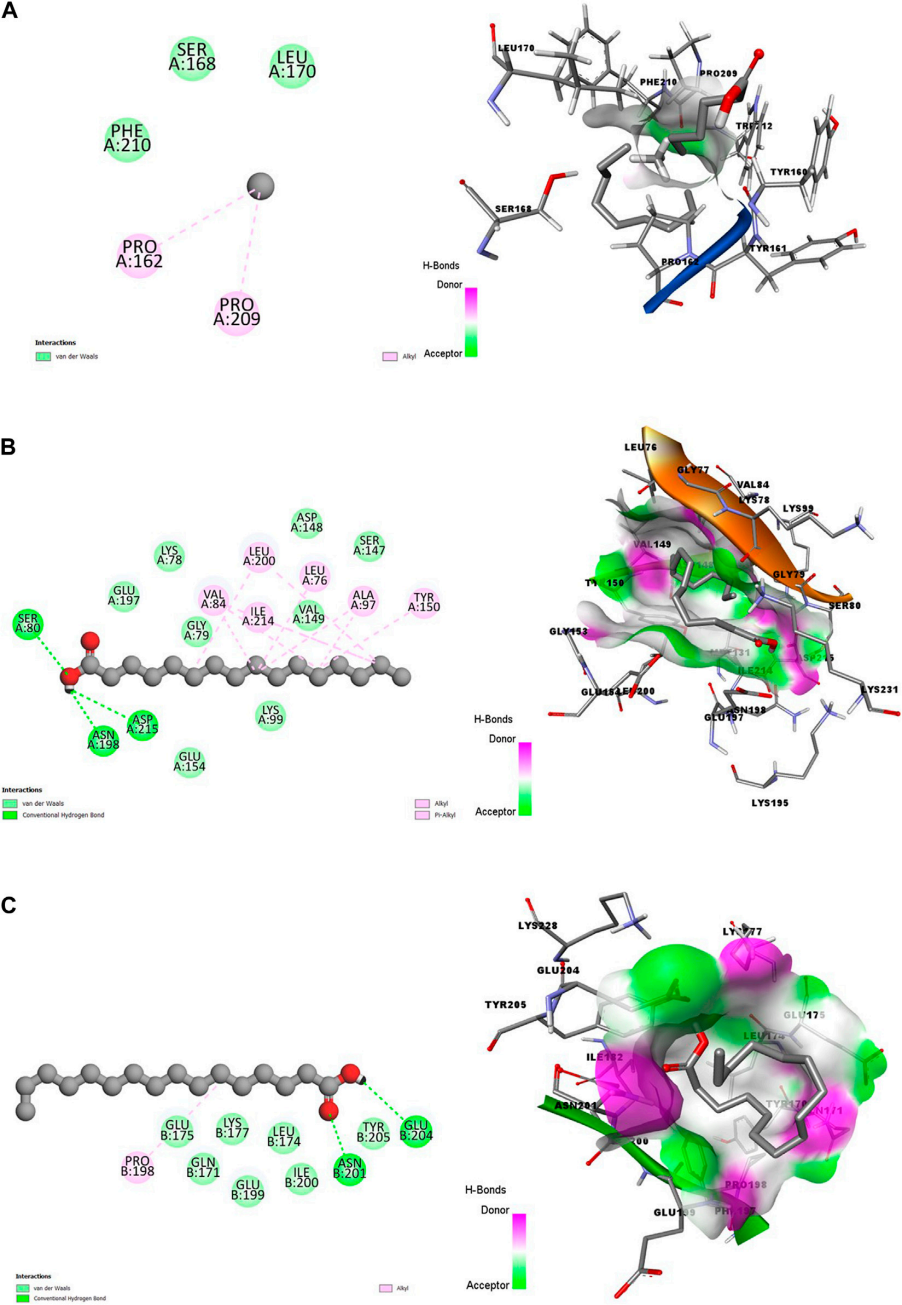
2D (left) and 3D (right) visuals of the molecular interactions between n-Hexadecanoic acid and amino acid residues of Asexual Blood Stage target proteins **(A)** Purine nucleoside phosphorylase **(B)** Calcium-independent protein kinases and **(C)** Dihydrofolate reductase-thymidylate synthase.

**FIGURE 2:**
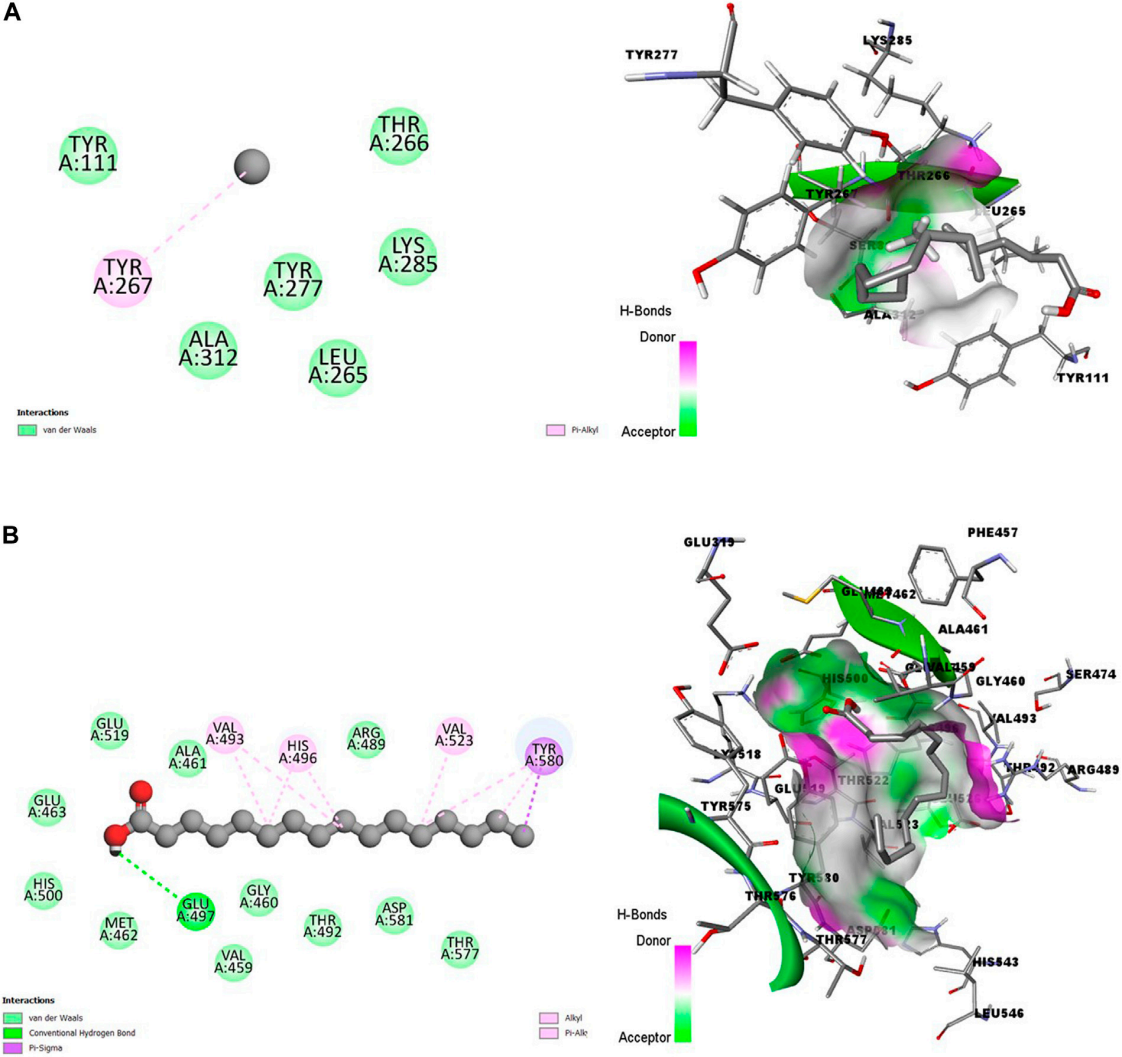
2D (left) and 3D (right) visuals of the molecular interactions between n-Hexadecanoic acid and amino acid residues of Hepatic Schizonts stage target proteins **(A)** Enoyl-ACP Reductase and **(B)** Ml alanyl aminopeptidase.

**FIGURE 3:**
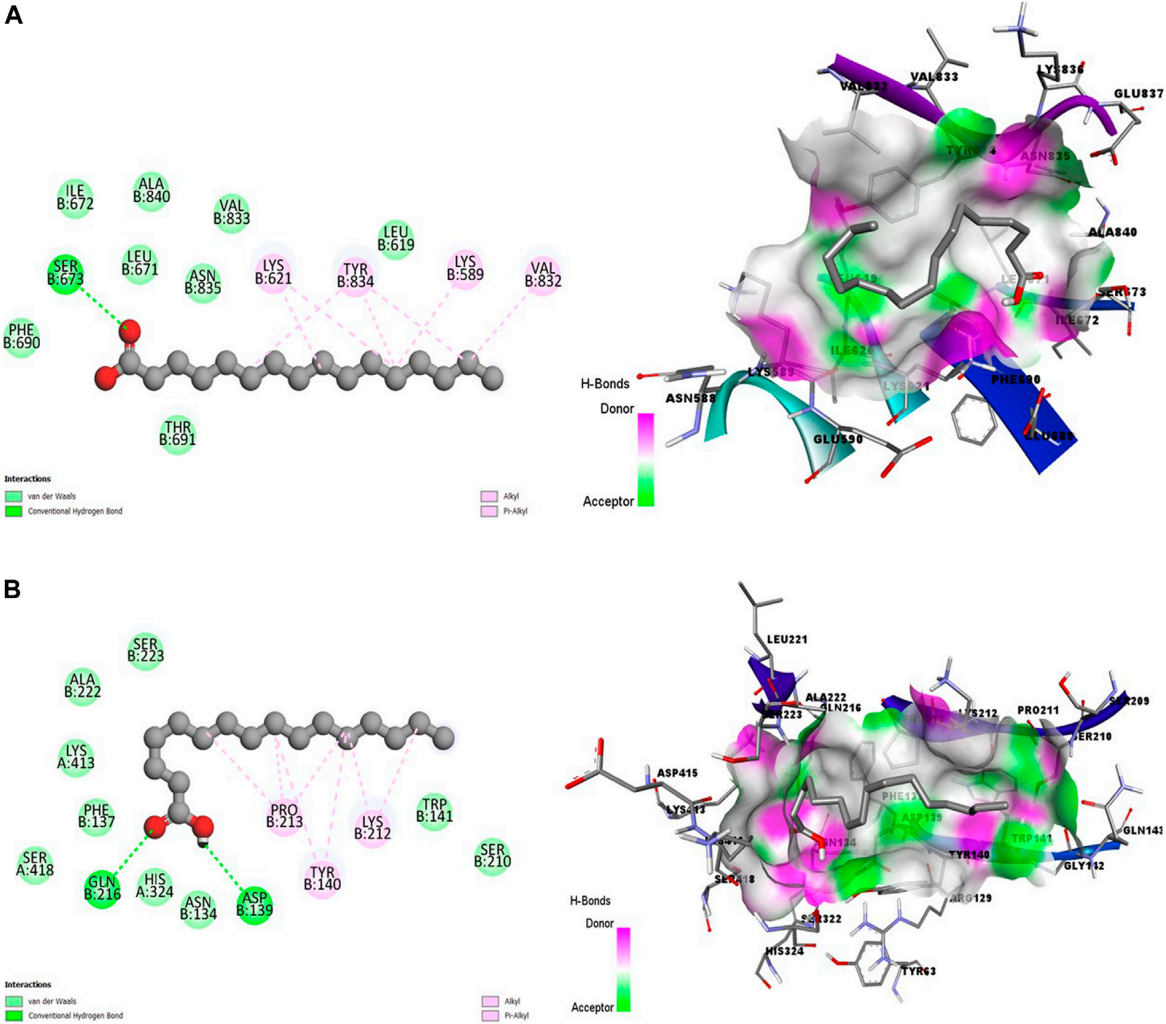
2D (left) and 3D (right) visuals of the molecular interactions between n-Hexadecanoic acid and amino acid residues of Transmission stage 1 (Targeting parasite gametocytes) Target Proteins **(A)** Cysteine-rich 230 kDa gamete surface protein and **(B)** Gametocyte surface protein.

**FIGURE 4:**
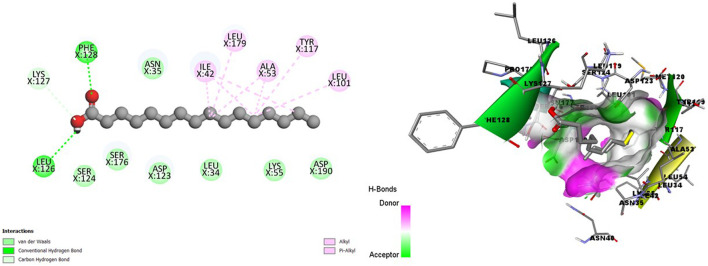
2D (left) and 3D (right) visuals of the molecular interactions between n-Hexadecanoic acid and amino acid residues of the Transmission stage (Targeting the insect vector) Target Protein, namely, Protein kinase 7.

In Figure 1–4, the 2-dimension and 3-dimension visual representations of the interactions between HA and the amino acid residues of the eight *Plasmodium spp.* target proteins are shown with each stage specified.

The 3D visuals of Figures 1A–C showed that the ligand (HA) is poorly inserted into the binding pocket of the PNP. Conversely, CDPK and DHFR-TS proteins had better interactions with HA, represented by hotspots of hydrogen bonds donor and acceptor well-represented in purple and green, respectively. Also, the two complexes formed hydrophobic interactions, including alkyl and Pi-Alkyl bonds. Out of the three, the PNP protein formed fewer interactions with the bioactive compound.

In Figures 2A, B, the poor state of interactions between the HA and the amino acid residues of ENR is visualized in 2D and 3D representations (Figure 2A). The poor state of interactions and insertions suggests poor stability of the receptor-ligand complexes. Conversely, Figure 2B shows multiple interactions between HA and amino acid residues of A-M1 protein, with the ligand better inserted into the binding pocket. There is also an abundance of conventional hydrogen bonds and hydrophobic interactions between HA and A-M1 compared to a single Pi-Alkyl between the HA and ENR protein.

Figures 3A, B showed promising coupling of the ligand-receptor complex between HA and the amino acid residues of s230 and s48/45 proteins. The interactions largely involve conventional hydrogen bonds and hydrophobic bonds, including Alkyl and Pi-Alkyl bonds.

Figure 4 showed a good interaction of the HA into the binding pocket of PK7 with favorable bonds, including conventional hydrogen bonds, carbon-hydrogen bonds, alkyl bonds, and Pi-alkyl bonds.

Clearly, a combination of the binding affinity and their interaction suggests that HA can inhibit at least a target protein in the four stages of the Plasmodium lifecycle, specifically CDK7(Asexual Blood stage), A-M1 (Hepatic Schizonts stage), s230 and s48/45 (Transmission stage 1) and PK7 (Transmission stage 2) as they formed promising complexes. This indicates a multi-stage-multi-target antiplasmodial potential of the HA.

### 3.3 Outcome of adsorption, distribution, metabolism, excretion and toxicity analysis

The pharmacokinetic and pharmacodynamics properties of HA were explored using various ADMET parameters, medicinal chemistry, and physicochemical properties, as shown in Tables 2–5. Table 2 shows that HA did not fail any of Lipinski’s rules of five based on its molecular weight, LogP value, number of hydrogen bond donors and acceptors, and Topological polar surface area, meaning a good absorption or permeability is possible. Also, the Graph Attention-based assessment of Synthetic Accessibility (GASA) suggests that synthesizing HA will be relatively easy, while a zero value in Golden Triangle indicates that the compound has a favorable ADMET property. Table 3 shows negative results for BBB penetration while it’s a non-inhibitor of Pgp, OATP1B3, and MRP1. Contrarily, n-Hexadecanoic is predicted to be capable of permeating Caco-2, and inhibition of OATP1B1 with negative result for human intestinal absorption. In Table 4, n-Hexadecanoic acid is predicted to be a non-inhibitor of Cytochrome P450 enzyme except CYP2B6 and CYP2C8. In terms of excretion, the compound is shown to have low clearance and ultra short half-life. Table 5 shows that n-Hexadecanoic acid is negative for multiple toxicity tests, except skin irritation and eye corrosion.

**TABLE 2:** The Selected Physicochemical Properties and Medicinal Chemistry Properties of n-Hexadecanoic Acid.

Compound	MF	MW		logP		nHA		nHD	TPSA	Lipinski rule	GASA	Golden triangle
n-Hexadecanoic acid	C_16_H_32_O_2_	256.24		6.648		2		1	37.3	0	0	0

Note: MF; Molecular Formula: MW; Molecular Weight: LogP; The logarithm of the n-octanol/water distribution coefficient at pH = 7.4: nHA; number of Hydrogen bond acceptor: nHD; number of Hydrogen bond donor: TPSA; Topological Polar Surface Area (Optimal: < 140): Lipinski Rule; MW ≤ 500; logP ≤ 5; Nha ≤ 10; nHA ≤ 5: GASA; Graph Attention-based assessment of Synthetic Accessibility (Probability of being difficult to synthesize from easy to hard - 0–1): Golden Triangle; 200 ≤ MW ≤ 500; −2 logD ≤ 5.

**TABLE 3:** The Selected Adsorption and Distribution Parameters of the n-Hexadecanoic Acid.

Compound	Caco-2 permeability	Pgp-inhibitor	HIA	F_50%_	PPB (%)	BBB	OATP1B1 inhibitor	OATP1B3 inhibitor	MRP1 inhibitor
n-Hexadecanoic acid	−5.096	0.0	0.85	0.438	98.031	0.021	No	Yes	Yes

Note: Caco-2, permeability; higher than −5.15 log unit is optimal: Pgp; P-glycoprotein (Category Inhibitor: Yes, Category Non-inhibitor: No): HIA; Human Intestinal Absorption (Category 1: HIA < 30% means positive, Category 0: HIA ≥30% means negative): F_50%_; 50% Oral Bioavailability (Category 1: F_50%_ < 50% means positive, Category 0: F_50%_ ≥ 50% means negative): PPB; Plasma Protein Binding (lower than 90% is optima): BBB; Blood Brain Barrier (Category 1: BBB positive; Category 0: BBB negative): OATPs; Organic Anion Transporting Polypeptides glycoprotein (Category Inhibitor: Yes, Category Non-inhibitor: No): MRP1; Multidrug resistance-associated protein 1 (Category Inhibitor: Yes, Category Non-inhibitor: No).

**TABLE 4:** The Selected Metabolism and Excretion Parameters of the n-Hexadecanoic Acid.

Compound	CYP1A2 inhibitor	CYP2C19 inhibitor	CYP2C9 inhibitor	CYP2D6 inhibitor	CYP3A4 inhibitor	CYP2B6 inhibitor	CYP2C8 inhibitor	CL_plasma_	T_1/2_
n-Hexadecanoic acid	No	No	No	No	No	Yes	Yes	Low clearance	Ultra-short

Note: CYP; Cytochrome P450 (Category Inhibitor: Yes, Category Non-inhibitor: No): CL_plasma_; Drug clearance ( > 15 mL/min/kg: high clearance; 5–15 mL/min/kg: moderate clearance; < 5 mL/min/kg: low clearance): T_1/2_; Half-life of drug plasma concentration to decrease by 50% (ultra-short half-life drugs: 1/2 < 1 h; short half-life drugs: T1/2 between 1–4 h; intermediate short half-life drugs: T1/2 between 4–8 h; long half-life drugs: T1/2 > 8 h)

**TABLE 5:** The Selected Toxicity Parameters of the n-Hexadecanoic Acid.

Compound	hERG blockers	DILI	Ames mutagenicity	Rat oral acute toxicity	Carcinogenicity	Skin irritation and eye corrosion	Human hepatotoxicity	Genotoxicity
n-Hexadecanoic acid	Negative	Negative	Negative	Negative	Negative	Positive	Negative	Negative

Note: hERG; the human Ether-à-go-go-Related Gene (Category Inhibitor: Positive, Category Non-inhibitor: Negative): DILI; Drug-Induced Liver Injury (Risk Category 1: Positive, Risk Category 0: Negative): AMES, Toxicity (Risk Category 1: Positive, Risk Category 0: Negative): Rat Oral Acute Toxicity (Risk Category 1: Positive, Risk Category 0: Negative); Carcinogenicity (Risk Category 1: Positive, Risk Category 0: Negative): Skin Irritation and Eye Corrosion (Risk Category 1: Positive, Risk Category 0: Negative): Human Hepatoxicity (Risk Category 1: Positive, Risk Category 0: Negative): Genotoxicity (Risk Category 1: Positive, Risk Category 0: Negative).

### 3.4 Outcome of suppressive antiplasmodial activity test and mean survival time

The n-Hexadecanoic suppresses the parasite load in a dose-dependent manner and is significantly different at *p* < 0.05 when compared to the negative control. The highest percentage of chemo-suppression was recorded from 100 mg/kg of HA (89.74%), showing higher chemo-suppression than Artemether/lumefantrine (88.38%). Also, the percentage chemo-suppression of HA-treated groups 50 mg/kg and 100 mg/kg are not significantly different from the standard drug, but 10 mg/kg of HA is significantly different when compared to Artemether/lumefantrine (Table 6). The mean survival time (MST) of the test groups at *p* < 0.05 was not significantly different when compared to the negative control, while the MST of the positive control group was significantly different at *p* < 0.05 (Table 6).

**TABLE 6:** Suppressive Antiplasmodial Activity and Mean Survival Time (MST) Of n-Hexadecanoic Acid on *Plasmodium berghei*-Infected Mice.

Treatment	Dosage (mg/kg)	Suppressive parasitemia (%)	Percentage chemosuppression (%)	MST (days)
HA	10	1.89 ± 0.30^ab^	71.58	15.60 ± 2.62^d^
HA	50	1.08 ± 0.29^a^	83.80	9.20 ± 1.16^d^
HA	100	0.68 ± 0.18^a^	89.74	11.40 ± 1.6^d^
A/L	2.5/13.5	0.77 ± 0.23^a^	88.38	26.00 ± 4.00^c^
NC		6.64 ± 1.68		12.60 ± 0.7

HA: n-Hexadecanoic acid; A/L: Artemether/lumefantrine; NC: negative control; n = 5; Data as Standard error of mean (±SEM).

^a^ Significant difference (*p* < 0.05) when compared with negative control.

^b^ Significant difference (*p* < 0.05) when compared with A/L.

^c^ Significant difference when compared with negative control.

^d^ No significant difference (*p* ≤ 0.05) when compared with negative control.

Reduction in body weight as well as temperature in the test groups and control groups were recorded, but their percentage gain in body weight (Table 7) and temperature (Table 8) were not significantly different before and after the experiment. The packed cell volume (PCV) of the test groups, as well as the Artemether/lumefantrine group, increased after treatment, and the negative control group had a loss of PCV on the terminal day (Table 9). The PCV was also checked on the tenth day after the commencement of the experiment; only the Artemether/lumefantrine group gained PCV of 1.6%. The PCV of the test group and negative control showed significant reduction *p* < 0.05 (Table 9).

**TABLE 7:** Effect of n-Hexadecanoic Acid on Weight of *Plasmodium berghei* Infected Mice.

Treatment	Dosage (mg/kg)	Weight (g)		Percentage change in weight (%)
		initial	final	
HA	10	22.88 ± 0.47	19.24 ± 0.81	−15.99 ± 2.63
HA	50	24.48 ± 0.71	22.94 ± 1.24	−6.46 ± 2.75
HA	100	22.19 ± 0.40	19.99 ± 0.31	−9.77 ± 2.56
A/L	2.5/13.5	23.80 ± 0.44	20.94 ± 1.07	−12.17 ± 3.38
NC		23.17 ± 0.31	21.29 ± 0.80	−8.05 ± 3.59

HA: n-Hexadecanoic acid; A/L: Artemether/lumefantrine; NC: negative control; n = 5; Data as Standard error of mean (SEM); No significant difference at *p* < 0.05.

**TABLE 8:** Effect of n-Hexadecanoic Acid on the Temperature of *Plasmodium berghei*-Infected Mice.

Treatment	Dosage (mg/kg)	Temperature (°C)		Percentage change in temperature (%)
		initial	final	
HA	10	32.26 ± 0.33	30.28 ± 0.24	−6.08 ± 1.60
HA	50	32.20 ± 0.32	30.08 ± 0.37	−6.56 ± 1.17
HA	100	32.20 ± 0.06	30.14 ± 0.46	−6.39 ± 1.59
A/L	2.5/13.5	31.94 ± 0.38	30.56 ± 1.27	−4.26 ± 1.47
NC		32.16 ± 0.25	30.22 ± 0.39	−6.00 ± 1.46

HA: n-Hexadecanoic acid; A/L: Artemether/lumefantrine; NC: negative control; n = 5; Data as Standard error of mean (SEM). No significant difference at *p* < 0.05

**TABLE 9:** Effect of n-Hexadecanoic Acid on Packed Cell Volume (PCV) of *Plasmodium berghei* Infected Mice.

Treatment	Dosage (mg/kg)	PCV (%)			Percentage change in PCV (%)	
		DAY 0	DAY 4	DAY 9	DAY 0	DAY 9
HA	10	42.63 ± 1.25	43.75 ± 5.11	23.12 ± 0.46	6.00 ± 13.99	−45.54 ± 3.26
HA	50	46.60 ± 2.88	48.20 ± 3.14	22.93 ± 4.57	3.54 ± 3.53	−51.35 ± 8.51
HA	100	40.94 ± 2.51	43.00 ± 5.03	25.42 ± 12.08	5.03 ± 2.78	−39.10 ± 10.67
A/L	2.5/13.5	40.94 ± 1.82	45.80 ± 2.82	42.44 ± 3.14	11.89 ± 4.82	1.60 ± 4.10
NC		42.82 ± 1.87	42.40 ± 1.44	27.83 ± 4.54	−0.71 ± 2.59	−35.07 ± 10.67

HA: n-Hexadecanoic acid; A/L: Artemether/lumefantrine; NC: negative control; DAY, 0: first day of infection; DAY, 4: the fifth day after infection; DAY, 9: the tenth day after infection; Data as Standard error of the mean (SEM). No significant difference at *p* < 0.05

### 3.5 Haematological parameters

There was no significant difference in the packed cell volume, red blood cells, haemoglobin, white blood cells, lymphocytes, neutrophils, monocytes, and eosinophil of the test groups and that of the control groups. A significant difference at *p* < 0.05 was seen in the platelet of mice treated with 5 mg/kg and 10 mg/kg of HA when compared with the control groups (Table 10).

**TABLE 10:** Haematological Parameters of uninfected, n-Hexadecanoic acid-treated mice.

Treatment	PCV%	HB(g/dL)	RBCs(x10^6 ^µl)	WBCs (x10^3^ μ**l)**	Platelet	LYM %	Neut %	Mon %	EOS %
HA2	49.00 ± 1.53	15.70 ± 0.47	8.01 ± 0.35	2,717 ± 622.0	48,667 ± 8,452	76.00 ± 0.58	21.33 ± 1.20	1.67 ± 0.88	1.00 ± 0.58
HA5	51.00 ± 4.00	16.40 ± 1.20	8.19 ± 0.42	3,217 ± 290.6	38,667 ± 5,696*	75.67 ± 0.88	21.67 ± 0.88	2.33 ± 0.33	1.33 ± 0.33
HA10	45.67 ± 0.67	14.80 ± 0.31	7.46 ± 0.04	3,067 ± 218.6	39,000 ± 1,528*	74.00 ± 0.58	22.33 ± 0.67	1.33 ± 0.33	2.33 ± 0.67
HA50	48.67 ± 4.06	15.67 ± 1.16	7.68 ± 0.62	3,200 ± 332.9	48,000 ± 6,351	74.67 ± 1.86	22.00 ± 0.58	1.33 ± 0.33	1.33 ± 0.88
HA100	51.33 ± 4.67	16.23 ± 1.32	8.07 ± 0.61	3,500 ± 256.6	4,900 ± 12,503	76.00 ± 1.00	21.33 ± 0.33	1.33 ± 0.33	1.33 ± 0.33
Control	46.67 ± 0.88	15.10 ± 0.38	7.67 ± 0.14	3,083 ± 44.10	90,000 ± 4,000	74.33 ± 1.20	23.33 ± 1.33	1.33 ± 0.33	1.00 ± 0.00

PCV: packed cell volume; HB: haemoglobin; RBCs: Red Blood Cells; WBCs: White Blood Cells; Platelet; NEU: neutrophils; LYM: lymphocytes; MON: monocytes; EOS: eosinophil; Data as the standard error of the mean (SEM); *significant difference when compared to control.

### 3.6 Histopathology analysis

**FIGURE 5:**
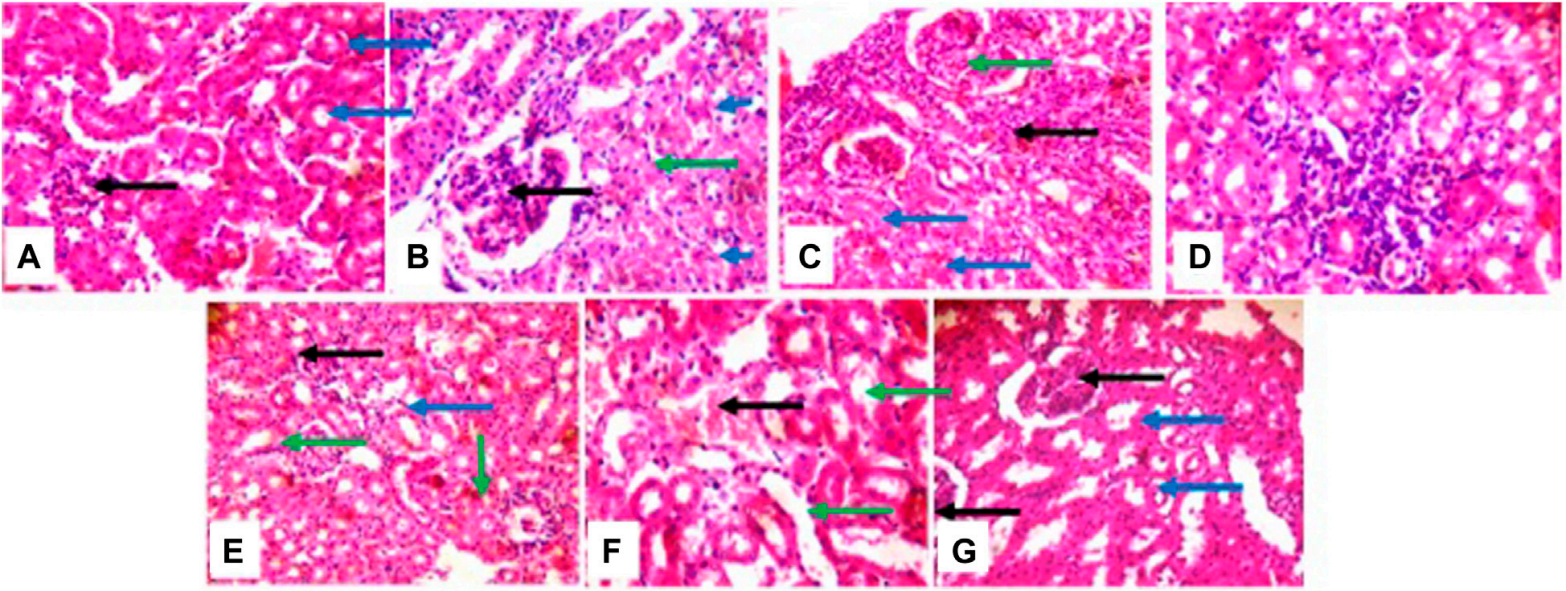
photomicrographs of the liver, (group **(A)**) and (group **(G)**) show normal architecture (group **(B)**) portal triads with inflammatory cells (green), hepatocytes show cytoplasmic vacuolation (blue). (group **(C)**) mild portal triads (green), liver parenchyma shows a large area of inflammatory cells aggregate and the sinusoids appear mildly infiltrated (black arrow); (group **(D)**) portal triads with mild peri infiltration of inflammatory cells (black), sinusoids appear mildly infiltrated (blue); (group **(E)**) and (group **(F)**) sinusoids show areas of mild to moderate inflammatory cells aggregate (black).

The liver histological presentation shows normal architecture in vehicle control mice (group a) and the positive control (group g) (cyclophosphamide) mice. However, mice treated with 2 mg/kg (group b) show central venules and portal triads with infiltration of inflammatory cells, hepatocytes show cytoplasmic vacuolation, and sinusoidal appears normal, not infiltrated. In mice treated with 5 mg/kg (group c), there are mild portal triads; hepatocytes appear normal, liver parenchymal shows a large area of inflammatory cells aggregate, and the sinusoids are mildly infiltrated. The mice treated with 10 mg/kg (group d) show central venules with mild to moderate perivascular infiltration of inflammatory cells, hepatocytes show cytoplasmic vacuolation, and sinusoids show focal areas of a mild aggregate of inflammatory cells. In mice treated with 50 mg/kg and 100 mg/kg (groups e and f), central venules and hepatocytes appear normal, and sinusoids show a focal area of mild to moderate aggregated infiltrated cells (Figure 5).

**FIGURE 6:**
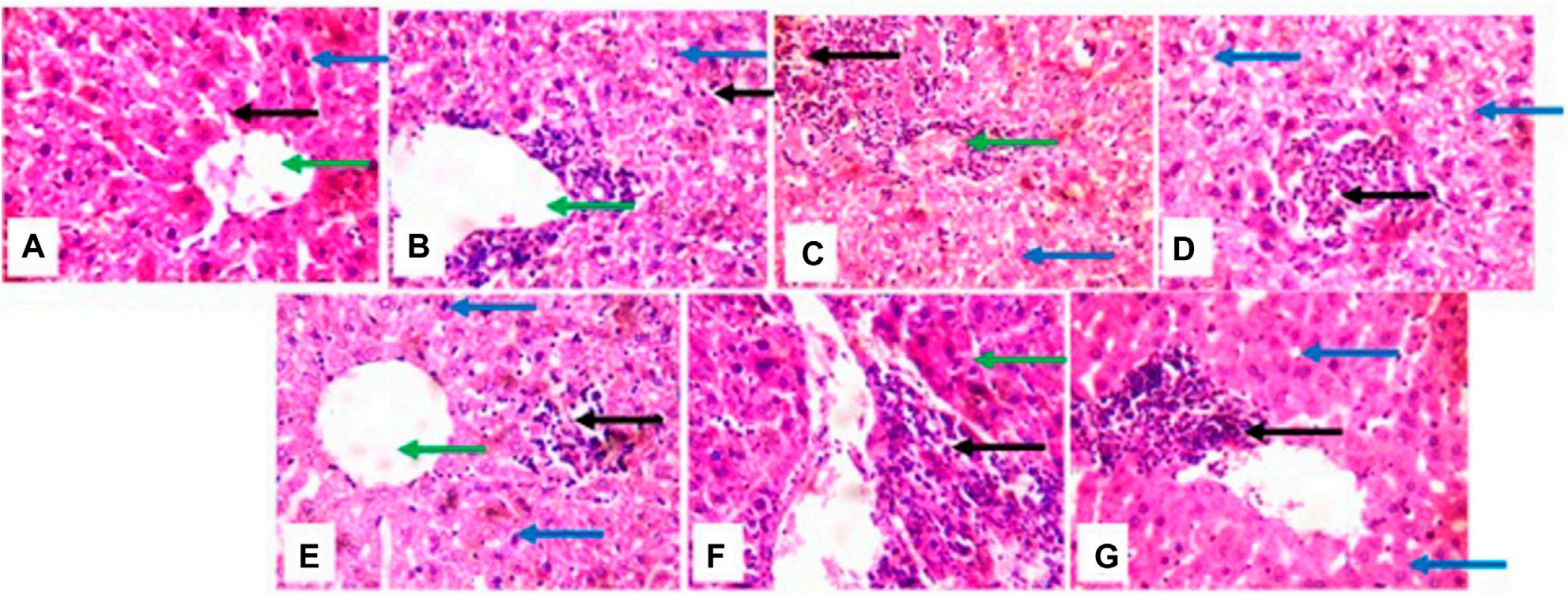
photomicrographs of kidney; (group **(A)**), (group **(B)**), and (group **(G)**) show normal architecture; (group **(C)**) renal cortex shows some glomeruli with peri infiltration of inflammatory cells (green arrow), renal tubule collapsed and luminar space are lost (blue arrow), interstitial spaces show area of moderate infiltration of inflammatory cells (black arrow): (group **(D)**) interstitial spaces show the focal area of inflammatory cells (black arrow) (group **(E)**) atrophic glomerulus (black). some of renal tubules show eosinophilic cast within the lumen (green arrow): (group **(F)**) renal tubule dilated (green arrow), interstitial spaces shows mild congestion (black).

The histological presentation of the kidney shows normal architecture in negative control mice (group a), positive control, cyclophosphamide mice (group g), and mice treated with 2 mg/kg of HA (group b). In mice treated with 5 mg/kg, 10 mg/kg, 50 mg/kg, and 100 mg/kg of HA, the renal cortex shows glomeruli with peri infiltration of inflammatory cells, glomeruli with fluid accumulation few with messengial hyperplasia, renal tubules appeared collapsed with the loss of luminar spaces. Also, some of the renal tubules show eosinophilic cast within the lumen, and interstitial spaces show areas of moderate infiltration of inflammatory cells and severe infiltration of inflammatory cells (100 mg/kg) (Figure 6).

### 3.7 Micronucleus assay

There was a significant difference in body weight of the cyclophosphamide treated mice group and 100 mg/kg of HA-treated groups when compared to the negative control group. There was no significant difference after the exposure and no significant difference in percentage weight gain except in the group treated with 100 mg/kg of HA (Table 11). The MnPCE frequency in bone marrow cells of mice exposed to HA was not significantly different at *p* < 0.05 in all treated groups when compared with negative control except in the 100 mg/kg BW and cyclophosphamide group. The percentage PCE: NCE was significantly different in all treated groups when compared to negative control. There is no significant difference in MnNCE in the test groups, only cyclophosphamide was significantly different at *p* < 0.05. Also, other abnormalities are not significantly different, but there was a significant difference in 100 mg/kg BW and cyclophosphamide groups (Table 12).

**TABLE 11:** Weight of experimental animals for toxicity test.

Treatment	Dosage (mg/kg)	Weight (g)		Percentage change in weight (%)
		Initial	Final	
HA	2	23.60 ± 0.75	23.91 ± 0.40	1.65 ± 2.94
HA	5	23.40 ± 0.25	22.56 ± 0.26	−4.65 ± 1.88
HA	10	21.60 ± 0.93	24.42 ± 1.39	12.84 ± 2.78
HA	50	23.20 ± 1.07	22.81 ± 1.74	1.04 ± 4.29
HA	100	27.20 ± 0.37***	27.10 ± 1.04	−19.65 ± 20.36*
CYP	20	24.53 ± 0.27 *	24.43 ± 0.25	−0.50 ± 0.34
NC		21.60 ± 0.25	24.77 ± 0.61	14.71 ± 2.94

HA: n-Hexadecanoic acid; CYP (Cyclophosphamide); NC: negative control; n = 5; Data as Standard error of the mean (SEM); * significant difference at *p* < 0.05 when compared to the negative control group; *** significant difference at *p* < 0.005 when compared to the negative control group.

**TABLE 12:** Toxicity of Hexadecanoic acid using Micronucleus assay.

Treatment	Dosage (mg/kg)	MnPCE	MnNCE	Others	PCE: NCE
HA	2	6.25 ± 0.88	3.42 ± 0.30	19.67 ± 1.58	2.32 ± 0.12*
HA	5	9.08 ± 1.30	3.17 ± 0.74	22.50 ± 1.77	2.21 ± 0.22**
HA	10	11.58 ± 2.00	6.67 ± 2.25	40.08 ± 1.31	1.44 ± 0.12***
HA	50	18.86 ± 1.74	7.19 ± 0.98	46.28 ± 2.22	1.11 ± 0.05***
HA	100	23.11 ± 0.58*	8.00 ± 0.84	56.78 ± 1.39*	0.82 ± 0.05***
CYP	20	225.9 ± 8.84***	100.9 ± 11.99***	119.2 ± 19.59***	0.51 ± 0.04***
NC		7.22 ± 1.28	3.89 ± 0.78	17.78 ± 2.00	2.84 ± 0.12

HA, hexadecanoic acid; CYP (cyclophosphamide 20 mg/kg/bwt); Negative control; micronucleated polychromatic erythrocytes (MnPCE); micronucleated normochromatic erythrocytes (MnNCE); other abnormalities (others); PCE: NCE (polychromatic erythrocytes/normochromatic erythrocytes ratio); *significant difference at *P* < 0.05 when compared to negative control; **significant difference at *P* < 0.01 when compared to negative control; ***significant difference at *P* < 0.005 when compared to negative control; Data as SEM.

## 4 Discussion

Several indigenous medicinal plants have been used to cure a variety of illnesses as well as other health benefits (Frimpong et al., 2021). More specifically, many plants have exhibited antimalarial activity and provide medicines for malaria treatment (Afolayan et al., 2016; Afolayan and Ijidakinro, 2021). One such plant is *Vernonia amygdalina* (Bitter Leaf), which has been previously reported to be used for treating malarial in folkloric medicine (Ugbogu et al., 2021). The most abundant phytocompound, n-Hexadecanoic acid (HA), in the bitter leaf, has been widely reported to have antimalarial properties (Bihonegn et al., 2019). Nevertheless, the stage of *Plasmodium spp.* lifecycle in which the bioactive is likely to have a significant effect, the chemosuppression performance *in vivo,* and its toxicity state remain unknown. To close these knowledge gaps, a combination of bioinformatics approaches and wet lab experiments are conducted in this study.

A combination of molecular docking analysis and visualization was employed to study the interaction of the bioactive HA with key therapeutic targets at four different stages of *Plasmodium spp*. lifecycle, including the asexual blood stage, hepatic schizonts stage, transmission stage (focusing on parasite gametocytes), and transmission stage 2 (targeting the insect vector) (Venugopal et al., 2020). An assessment of the binding affinity and ligand-receptor interaction of the bioactive with selected targets of each stage indicates promising therapeutic interactions. n-Hexadecanoic clearly formed a favorable complex with at least a therapeutic target protein in each stage and, more importantly, interact with amino acid residues in the active sites, including calcium-independent protein kinases and hihydrofolate reductase-thymidylate synthase proteins (asexual blood stage); cysteine-rich 230 kDa gamete surface protein and Gametocyte surface protein (gametocyte stage); Protein Kinase 7 (insect vector stage); and M1 alanyl aminopeptidase (Hepatic Schizonts stage).

These proteins are validated drug targets that play crucial roles in the survival, growth and transmission of the parasite. For instance, *Plasmodium falciparum* dihydrofolate reductase-thymidylate synthase (PfDHFR-TS) are common targets for antifolate drugs, including cycloguanil and pyrimethamine. The PfDHFR-TS is vastly involved in the production of folates and generates thymidylate, which is needed for the pathogen DNA synthesis (Chaianantakul et al., 2013). The inhibition of the DHFR domain of PfDHFR-TS through binding to the active site of the enzyme helps hinder the conversion of DHF to tetrahydrofolate, resulting in disruptions in DNA production and cell death of the pathogen (Hyde, 2005). At the gametocyte stage, cysteine-rich 230 kDa gamete surface protein (PF230) is expressed abundantly on the surface of mature female gametocytes, the sexual stage of the parasite within the human host. During a blood meal by a female mosquito, these gametocytes are taken up and differentiated into male and female gametes. PFS230 on the female gamete surface interacts with a sperm protein on the male gamete, facilitating sperm binding and initiating fertilization (Ali et al., 2021). The development of small molecules is one of the key approaches in disrupting the binding between PF230 and the sperm protein. In other words, they block fertilization without causing harm to the host (Consalvi et al., 2022). Challenging the insect vector by inhibiting the Protein Kinase 7 is also possible as a therapeutic solution. The *Plasmodium falciparum* Protein Kinase 7 (PfPK7) plays a key role in intracellular signaling pathways, which are needed for parasite development. Consequently, researchers have been exploring small molecules to inhibit PFPK7 to disrupt the essential roles, which the protein is involved with (Adelusi et al., 2023). Also, the inhibition of M1 alanyl aminopeptidase can help suppress the burden of the Plasmodium pathogen at the liver stage, as the protein is involved in hemoglobin cleavage within the parasite. The inhibition of PFA-M1 stops hemoglobin digestion and, consequently, disrupts the survival of the parasite (Hoff et al., 2021).

This suggests the potential of the HA as an antiplasmodial agent capable of targeting multiple targets and stages. Given the increasing mutations and resistance by *Plasmodium spp.* in the fight against available drugs, having a drug agent capable of targeting multiple proteins is an important development. This is because multi-targeting drugs have been reported to generate better clinical response, with more drugs being developed to target multiple proteins (Fu et al., 2017; Makhoba et al., 2020). Compounds capable of modulating different targets continue to take center stage as they reduce the chances of drug resistance and are often suitable for the development of new hybrid drugs (Tibon et al., 2020).

To further validate the antiplasmodial potentials of HA, an *in vivo* study was carried out using a murine model. *Plasmodium berghei* was selected since it is widely available and regularly used to evaluate prospective antimalarial medicines in rodent models. One of the most often used techniques for evaluating possible antimalarial medicines is the 4-day suppressive test, which assesses a test compound’s efficacy on early malaria infection (Belay et al., 2018). This study shows the percentage of chemosuppression in a dose-dependent manner; the highest percentage of chemosuppression is seen in the group treated with the highest dose, which is higher than the standard drug. According to the literature, antiplasmodial activity can be classified as moderate, good, and very good if the test compound shows a percentage of chemosuppression equal to or greater than 50% (Fentahun et al., 2017). Thus, HA is an excellent antiplasmodial drug lead as it showed very good chemosuppression even at the lowest dosage of 10 mg/kg in this study. Also, the outcome of this study is in line with the findings of Omoregie and Pal, who reported that ethanol extract of *Vernonia amygdalina* has antiplasmodial activity against *P. berghei*-infected mice in a dose-dependent manner (Omoregie and Pal, 2016).

Furthermore, the mean survival time was not significantly different in the test group when compared with the negative control but was significant in the standard drug control group. The decrease in weight of all experimental animals may be the effects of the parasite on their body because there is a loss of appetite after inoculation. The change in temperature may be the impact of environmental factors. In this study, data from Days 4 and 9 were reported because they enabled us to gauge the early and sustained effects of the n-Hexadecanoic acid and control drugs on the Plasmodium berghei-infected Mice. Also, the percentage change was assessed to quantify the impact of the bioactive on the parasitemia during the critical period (Omoregie and Pal, 2016). The Packed Cell Volume (PCV), which measures the number of red blood cells in each whole blood and is used for assessing anemia or polycythemia (Oladejo et al., 2024), was checked on D9 of the experiment, and it reduced drastically in all groups except the standard drug group that slightly reduced. The reduction in PCV could be the result of hemolysis undergone by the red blood cells after the administration of some antimalarial drugs or as a result of Glucose-6-phosphate dehydrogenase deficiency; this deficiency serves as a protective measure for populations exposed to malaria (Brusick et al., 2016).

To assess the toxicity of HA, *in silico* prediction, histopathology analysis, and a micronucleus assay were used. This is important to determine the dose-response, potency, and safety of the compound for human use (Gupta et al., 2022). The bioactive is predicted to have favorable adsorption, distribution, metabolism, and excretion properties as it passes Lipinski’s rule of five (Haritha et al., 2024) and the Golden Triangle test (Zerroug et al., 2019). Also, the GASA prediction suggests that it is relatively easy to synthesize HA, which can translate into a cheaper cost if eventually approved as a marketable drug. More importantly, HA is predicted to be relatively safe within the body system as it is a non-blocker of the human ether-a-go-go gene and negative for drug-induced liver injury, rat acute toxicity, carcinogenicity, and genotoxicity test. Blocking the hERG can lead to its disruption, which can result in arrhythmias and potentially fatal heart conditions (Robertson et al., 2005). However, appropriate encapsulation of the drug lead may be necessary as it is predicted with the potential to be corrosive to the eyes and irritating to the skin.

However, given that a previous toxicological study conducted on *V. amygdalina* showed significant mortality and tissue damage in the freshwater snail *Bulinus truncates* (Eze et al., 2020), subacute toxicity tests covering several hematological parameters were conducted further to assess the safety profile of the bioactive compound. More so, it is crucial to assess the safety of HA at different dose levels to determine a lethal and non-lethal concentration. The liver and kidney are the main targets of toxic substances (Manaharan et al., 2014), as the disruption in the normal architecture of the liver and kidney acts as pointers of acute immune response to toxicity. In this study, the liver histological presentations inform that HA is capable of initiating inflammatory responses within the liver. This aligns with the immunomodulatory activities of V*. amygdalina* reported by Omoregie and Pal (Omoregie and Pal, 2016). However, lower dosages of 2 mg/kg, 5 mg/kg, and 10 mg/kg would be ideal as keeping a balance between pro-inflammatory and anti-inflammatory cells is crucial to the use of immunomodulatory therapy against malaria (Popa and Popa, 2021). More so, the results suggest that a higher dosage leads to higher infiltration of inflammatory cells and aggregation of sinusoids. Interestingly, kidney histopathology presentation suggests that HA is safe at a dosage of 2 mg/kg with the renal tubule looking rigid, luminar space present, and absence of inflammatory cells within the renal cortex, showing similar to the positive control and negative control. Additionally, the micronucleus assay has shown that HA is cytotoxic at 100 mg/kg and 50 mg/kg BW, which damages the chromosomes in the dividing cells of the exposed animals. As the concentration increases, the appearance of micronucleated polychromatic erythrocytes (MnPCE) and other abnormalities increases in the test groups. At higher concentrations, some of the cells appeared as apoptotic and necrotic cells (more cell damage was seen). This could be the result of lipid peroxidation, a process whereby lipids are oxidatively degraded and release free radicals (Ayala et al., 2014); these free radicals in the cell attract electrons from the cell membrane’s lipid, which leads to cell damage. The end-products of lipid peroxidation, such as malondialdehyde (MDA) and 4-hydroxynonenal (HNE), are biomarkers of lipid peroxidation, whose effect is similar to that of reactive oxygen species (Zarkovic, 2003). Also, these end-products may be mutagenic or carcinogenic (Postma et al., 1996). For instance, MDA reacts with deoxyadenosine and deoxyguanosine in the DNA, forming DNA adducts that may lead to the proliferation of abnormal or damaged red blood cells. Thus, a combination of these findings indicates that HA has antiplasmodial activity at four different stages of the Plasmodium lifecycle but in a dose-dependent manner with a low dosage of about 2 mg/kg to 5 mg/kg best advised. Nevertheless, it is recommended to conduct further biochemical studies on the safety profile and confirmatory study on whether HA can work synergistically with other approved drugs or promising antimalarial small molecules.

## 5 Conclusion

This study showed the promising multi-stage, multi-target antiplasmodial potentials of n-Hexadecanoic in a dose-dependent manner on *Plasmodium berghei*-infected mice. While the antiplasmodial activity of the bioactive from *Vernonia amygdalina* performs better at a higher dosage of 100 mg/kg with 89.74% chemosuppression, a lower dose between 2 mg/kg and 10 mg/kg is recommended for safety reasons based on multiple toxicity tests.

## Data Availability

The original contributions presented in the study are included in the article/Supplementary Material, further inquiries can be directed to the corresponding author.
